# Response: “Commentary: A Hypothesis for Examining Skeletal Muscle Biopsy-Derived Sarcolemmal nNOSµ as Surrogate for Enteric nNOSα Function”. nNOS^skeletal muscle^ may be Evidentiary for Enteric NO-Transmission Despite nNOSµ/α Differences

**DOI:** 10.3389/fmed.2016.00004

**Published:** 2016-02-22

**Authors:** Arun Chaudhury

**Affiliations:** ^1^Arkansas Department of Health and GIM Foundation, Little Rock, AR, USA

**Keywords:** skeletal muscle, membrane nNOS, active site, splice, nitrergic, biomarker, biopsy

Accommodation of food/liquid bolus or chyme/chyle within the esophagogastrointestinal lumen necessitates relaxation of the muscular wall (1–3). This relaxation is mediated by two neurotransmitters released in tandem from the nerve terminals that are in microscale proximity to the smooth muscles. Sequential evoked release of soluble purine derivatives, notably ATP and *de novo* synthesized gaseous nitric oxide (NO) relaxes the smooth muscles (4, 5). The mechanical relaxation is preceded by an electrical event, the inhibitory junction potential (IJP), which is the *sine qua non* of enteric musculomotor inhibitory neurotransmission (6–8).^1^ Regional movement also necessitates contraction of the smooth muscles mediated by cholinergic muscarinic stimulation but throughout the gastrointestinal tract, “inhibitory neurotransmission-mediated accommodation” is the single most important functional component that allows oro-aboral accommodation and progression of gut contents (9). Not surprisingly, the disorders pertaining to gastrointestinal motility mainly results from defects in accommodation, chiefly due to impairment of inhibitory neurotransmission (1, 10–12).

Importantly, the dual cascade of inhibitory neurotransmission posits a fascinating cell biology challenge. While the purine derivatives, including ATP, are released from membrane-delimited vesicles, nitric oxide (NO) is synthesized *de novo*, meaning that synthesis is instantaneously coupled to an incoming action potential at the nerve terminal^1^ (1). ATP-mediated fast IJP and NO-mediated slow IJP are coupled to each other in time, and this has been demonstrated by several labs over the decades and mires in no controversy. Theoretically, it may seem that nitric oxide may be synthesized by nNOS anywhere within the cell provided it has the right molecular arrangement, namely, a dimeric state and stimulus for electron flow for oxidation of l-arginine. However, surprisingly, in both neuronal and non-neuronal cells, nNOS appears to be located to specific cellular microdomains for activity. These microdomains are mainly localized at the cell membrane (13–15). Though detailed experiments to address the rationale of these cellular organizations are lacking, it is likely that the major purpose for specific microdomain localization of nNOS at the membrane may necessitate from the need to molecularly interact with calcium channels and other regulators of its activity (for example, specific kinases and phosphatases) (1). These allosteric mechanisms can only occur if macromolecular complexes are in enough nanoscale proximity within the cell. Here, it may be emphasized that the membrane-localized nNOS is possibly not the only site of nNOS presence. In almost all cells in which nNOS are present, staining for nNOS is diffusely seen within the cell. In the enteric nerve terminals, nNOS, despite its presence in the cytosol, does not actively synthesize NO. This is mainly because of its phosphorylated status, which renders it inactive for nitric oxide biosynthesis (15–17). However, particulate fraction of nNOS is the main active form has been demonstrated by different methodologies for nNOS distribution, including within enteric nerve terminals and skeletal muscles (18–26).

Herein belies the major significance of examining membrane-bound nNOS to test the efficiency of nitrergic neurotransmission. Despite scant attempts to obtain full-thickness intestinal biopsies, largely, this is not a common practice for the sheer challenge of perforating the gut. Thus, obtaining information from biomarkers is a major diagnostic issue and of paramount importance to the field of neurogastroenterology. In most gastrointestinal motility disorders, functional evidence of failure of relaxation is obtained indirectly by imaging and manometry (2, 11, 12). Preclinical studies reveal deficits of nitrergic function both in disorders involving the proximal and distal gut. However, an important dilemma arises while examining pathological specimens obtained from GI motility disorders. In these biopsies, nNOS staining appears normal, including in the enteric nerve terminals. These observations create confusion in clinicopathological diagnosis. A recent study has demonstrated that in diabetes mellitus, nNOS synthesis and distribution remain relatively unaffected, whereas myosin Va genomic expression is severely impaired, with virtual absence in the nerve terminals (27). Earlier, we had demonstrated the critical role of myosin Va in transport of nNOS in the enteric nerve terminals, possibly by interacting with LC8, the light chain of dynein (also called PIN, protein inhibitor of nNOS) (28). These findings may help clarify molecular targets for motility disorders, which are currently only classified as “idiopathic” or “functional.”

Using an inbred strain of mouse that lacks myosin Va, the dilute DBA/2J mice, we have additionally demonstrated that cavernosal nitrergic neurotransmission is impaired in deficiency of myosin Va (29). The major isoform that mediates nitrergic neurotransmission in the penile cavernosal nerve terminals is not nNOSα but rather nNOSμ (30), the same splice variant present in skeletal muscles. This study provided evidence of binding of nNOS and myosin Va in cavernosal extracts (29). Based on this background information, I presented my opinion that imaging and subcellular examination of skeletal muscle biopsies may potentially provide information on nNOS cellular localization within the enteric nerve terminal (31).

Though I am being repetitive, I shall elaborate why my opinion is rationally derived. Skeletal muscle contains nNOSμ, a closely related splice variant to nNOSα (32). Several groups have described the presence of nNOSμ and, in fact, highlighted that activity of nNOS is comparable in skeletal muscles than in the brain, despite tissue-specific differential translation. In a classical experiment, Lainé and de Montellano demonstrated that addition of nNOSμ to brain supernatant or pellet results in same levels of NO production as addition of nNOSα (33). The reciprocal experiment, addition of nNOSα to the leg muscle supernatant or pellet also yielded similar results to those obtained by addition of nNOSμ. These experiments could not discern differential tissue effects on the catalytic rates of the two isoforms (33). Thus, despite measurable differences (34), nNOSμ and α are very closely related to each other, not only molecularly but also in their catalytic properties. The subtle electronic properties may contribute to region-specific needs, but currently the basis is far from known. Most importantly, there can be no denial that most nNOS in skeletal muscles is clustered and confined to the space just below the cell membrane (32). This is a striking light microscopic appearance whenever one visualizes a skeletal muscle section stained for nNOS. Diffuse staining is seen throughout the sarcoplasm but is always much lighter in intensity. Thus, the nNOSμ at the cell membrane stands out in sharp contrast to nNOSμ diffusely spread within the cell cytoplasm. NADPH diaphorase examination reveals staining at the sarcoplasm, again suggesting that the nitric oxide synthesis is mainly confined to the periphery in skeletal muscles. In my perspective (31), I pointed out that this simple imaging observation may serve as a preliminary surrogate assay to examine the efficiency of cell membrane localization of nNOS in general and nNOSα in particular.

I never interpreted, neither posited, that nNOSμ is solely located at the cell membrane (31). There are important subcellular localizations of nNOS within the cytoplasm (26, 32, 35), with nNOSβ predominantly distributed in the cytoplasm and have been shown to be in association with Golgi complex (36). The discussion of nNOSβ is beyond the scope of this response, but I may briefly mention that even on this aspect, there is an intriguing similarity between the skeletal muscle and the enteric prejunctional nerve terminal. In the enteric terminal, nNOSβ is concentrated in the cytosol and remains phosphorylated; in contrast, however, to skeletal muscle, nNOSβ^ser847P^ does not produce NO when stimulated *in vitro* (16, 17). Interestingly, similar observation for nNOSβ has been recently reported in cardiomyocytes; nNOSβ are phosphorylated and remain in association with the cardiomyocyte cytoskeleton within the cytosol (37), similar to skeletal muscles (36). nNOSβ has the full enzymatic capacity to support oxido-reduction to generate NO but lacks the N terminal specialized domains to associate with membrane (31, 36). In skeletal muscles, nNOSβ may associate with dystrophins (36, 38). It merits further investigation whether availability of nNOSβ may be dependent on membrane localization to the subcellular organelle or can function in free-floating form, and what special adaptations it may need under such circumstances. Increasing evidence shows that NO biosynthesis may not be stochastic but rather rely on specific cellular microdomain(s) localization in order to have a functional allosteric relation (e.g., association with calcium channels on the ER or plasma membrane). In the general commentary, the author provides an erroneous reference of the role of cytoplasmic nNOS in the maintenance of RyR of endoplasmic reticulum (39). However, the study referenced is an *in vitro* study and does not specifically examine the contribution of membrane or cytoplasmic nNOS (39), and it merits specific techniques to knockout membrane-bound nNOS and thereafter assesses the role of nNOS localized elsewhere in the cell and its specific impact on physiological functions. There are other splice variants similar to nNOSβ, for example, the testicular nNOS (^Tn^nNOS), which shows a highly interesting molecular property (40). In built into its structure is the LC8 domain, which may aid in its localization to specific cellular domain. Further studies may help reveal the specific roles of nNOSβ in enteric nerves and skeletal muscles, including whether they act as a reserve when nNOSα does not function properly within the enteric or cavernosal nerves (35), as in chronic conditions such as diabetes mellitus, or the specific role of nNOS splice variants on genomic expression of local growth factors and impact on local or systemic organs (41–45).

Based on these observations, I hypothesized that in gastrointestinal motility disorders, including in idiopathic disorders, membrane-bound nNOS in sarcolemma may be reduced or deficient, as the intracellular transport mechanisms may be similar for nNOS in diverse cells and may depend on LC8/PIN and actin-interacting myosins, including myosin Va (1, 31). This was based on the assumption that myosin Va defect may be a major component of the motility disorders and could be distributed pathophysiological event. This was based on the common pathognomonic feature from available evidence that nNOS staining/expression and distribution in enteric nerve terminals remains relatively unaffected (including some reports mentioning increase in nNOS expression) in several motility disorders (46, 47), despite consistent observations of impaired nitric oxide synthesis in physiological experiments (27). Thus, skeletal muscle nNOS may be tested to obtain valuable information about different aspects of nNOS within enteric nerve terminals based on the following similarities:

(i) Genomic synthesis mechanisms and expression status. Though alternate splicing mechanisms contribute to the differential expression of nNOSμ and α (48), μ isoform is not exclusively present in skeletal muscles. For example, cavernosal nerves and several brain regions contain μ (30, 49). Similarly, though nNOSμ is the major transcript, some studies have suggested that nNOSα may also be present in skeletal muscles (50). This important point needs further validation. A very interesting aspect regarding nNOSμ and α transcriptional/translational similarity is demonstrated by the fact that in nNOSα knockout mice, nNOSμ is absent in skeletal muscles (36, 51). The nNOSα mice show prominent gastrointestinal symptoms, including notable gastroparesis on a solid diet (52) (Figure 1). This may severely affect their nutrition, thus contributing to the observed low muscle weight (36, 51). Using double knockout involving both nNOSα and β, preliminary studies have suggested lack of force development post-mild exercise regimens, and this has been attributed to the function of beta splice variant (36). This study also makes the important suggestion that sustained NO provides important feedback physiological impact to the skeletal muscle myofilaments (36). However, additional studies are needed to decipher the contributing role of NO synthesized by nNOSμ at the periphery, as well as novel models such as nNOSα + nNOSβ− mice to study the role of nNOSβ in mediating this function of force generation after a cycle of contraction–relaxation. In nNOSα knockout animals (36, 52), nNOSα may be absent in regions of skeletal NMJs and may impact postsynaptic acetylcholine receptor clustering. This may have important impact on force generation in the skeletal muscles, apart from chronic ischemia due to nNOSμ deficiency at the sarcolemma. Skeletal muscles may be useful for testing nNOSα/β stoichiometry and any commonality or differences in relation to their distribution in enteric nerve terminals. The suggested biopsies (31) may also provide important information regarding the regulation of nNOS transcription/translation and participatory genome regulatory elements for nitrergic biosynthesis and its allosteric modulators.
(ii) Mechanism of intracellular transport to the site of active nitrergic synthesis. The pools exchange. In the skeletal muscle, the nucleus is located in the periphery. Thus, after nNOS transcript synthesis, nNOSμ/α/β mRNA must be transported out of the nucleus and thence to the ER and Golgi for functional secretion back to the cytoplasm. A major portion of this pool gets actively localized to the sub-sarcolemmal region. Myosin Va may transport nNOSμ. Myosin Va has been shown to be present in skeletal muscles (31). Furthermore, it has been suggested that PIN/LC8, the adaptor of nNOS for myosin Va (27, 28, 31), is upregulated in skeletal muscles and is localized to the periphery in the sub-sarcolemmal region in Duchenne dystrophy (55). Despite the lack of dystrophin or nNOS^membrane^ in these muscles, upregulation of PIN likely results from compensatory mechanism due to lack of nNOS, suggestive of a role of PIN/LC8 in transport of nNOS to the peripheral sub-sarcolemmal region in skeletal muscles (55). The docking/clustering mechanisms for nNOS in the submembranous zone of the cell periphery are different in the skeletal muscles and in the enteric nerve terminals. Unlike the misrepresentation in the general commentary (56), I never posited that the membrane loose-adhesion mechanisms are same (31)! In the enteric nerve terminals, which are prejunctional in its anatomic localization, PSD95 tethers nNOS to the membrane (15), much like the postsynaptic cells of the central nervous system. The proteins involved are different in skeletal muscles and comprise macromolecular complexes of syntrophins, dystrophin, dystrobrevin, and sarcoglycans (32). However, there may be a lot of heterogeneity in these aspects. First of all, these anchoring mechanisms have been only limitedly studied in the ENS. Furthermore, many skeletal muscles may not have the complete repertoire and yet function, for example, chicken and turtle muscles do not have sarcoglycan (whereas, sarcoglycan deficiency causes major disease in humans) (57). Dystrophins and syntrophins may also be present in the CNS neurons (58). It is being gradually appreciated that in the face of molecular crowding (59), be it in the skeletal muscle or the enteric nerve terminals, precise chemical docking mechanisms due to molecular homologies of protein interactions potentially lead to precision in cellular streaming and localization and function, despite the spatial cytosolic molecular entropy (1, 60). This also contributes to a dynamic pool, exchanging nNOS between the membrane and cytosol with respect to specific physiological demands (1, 16, 32). Though the impact of pharmacological treatment is virtually unknown, one preclinical study has demonstrated that the gene expression of myosin Va is enhanced upon treatment of mdx mice with prednisolone (61). Thus, the comparative examinations of the splice variants acquire great significance. Interestingly, a recent proteomic study has demonstrated that in ­prednisolone-induced skeletal muscle myopathy, associated with membrane nNOS mislocalization, several classes of myosin heavy chains are upregulated, including Myh1, Myh2, Myh4, and Myh11 (62). This could likely be a compensatory mechanism to the loss of sarcolemmal nNOS, and the role of specific cortical myosins and its impact on nitrergic biosynthesis is only gradually being appreciated.
(iii) nNOSμ at the sarcolemma dislocates in skeletal muscle diseases, as well as systemic diseases in which gastrointestinal motility impairment manifest and may not necessarily be associated with induction of skeletal atrophy. In several primary skeletal muscle diseases, including Duchenne dystrophy, nNOSμ dislocates (32). This is an established finding. The sub-sarcolemmal dislocation leading to diffuse cytosolic staining are seen not only when membrane clustering mechanisms are deficient but also in myopathies, experimentally induced myasthenia, as well as in metabolic disease like diabesity (31, 32, 55, 62, 63). Given the role of NO in modulation of acetylcholine receptors (54, 64) (Figure 1) as well as the critical function of glucose uptake and insulin receptor functioning in the skeletal muscles (65, 66), it is reasonable to hypothesize that cytosolic transport of nNOSμ to the membrane is of tremendous significance and merits detailed examination. There may be some GI disorders, for example, cirrhosis-associated sarcopenia, which may result in chronic wasting and muscle fatigue, nNOSμ unloading and upregulation of autophagy (67), similar to muscle disease in COPD (68) or other disorders, such as critical illness myopathy, multiple sclerosis, cancer cachexia, chronic kidney disease, or cardiac cachexia (53, 54, 69, 70), but I had not focused on these polyvariable conditions in my earlier perspective (31). My main aim of the discussion remained whether we may find additional evidence of idiopathic motility disorders by examining skeletal muscle nNOS expression and distribution, given the practical difficulties of obtaining whole-thickness gut biopsies. In fact, not all skeletal muscle disorders in which nNOSμ are dislocated from the membrane generates an autophagic or atrogin/MuRF response. A recent study incorporating 164 skeletal muscle biopsies from different cohorts of patients, including skeletomuscular disorders, have shown that in nearly one-third of subjects, there is sub-sarcolemmal dislocation of nNOS but apparently without any neuromuscular problems (53, 62) (Figure 1). nNOSμ may be dislocated during spaceflight or microgravity simulation, but does it toggle between membrane and cytosolic location during relative periods of inactivity and hypotonia like sleep? Sarcolemmal nNOS is a common phenomenon observed across species (71). Similarly, it shall be interesting to examine the effects of physiological inactivity like hibernation and torpor on sarcolemmal nNOS distribution and recovery, or in subclinical conditions like chronic statin use, hypothyroidism, and HIV infection prior to development of frank cachexia. In general, nNOS localized to the membrane is protected from calpain-mediated autolysis (33, 72, 73); however, the precise mechanisms of timing of half-life of nNOS with respect to its cellular location are far from known, but definitely is another important area for exploration. Precise impact of functional gastrointestinal disorders (FGIDs) like irritable bowel syndrome (IBS) on nutrition and skeletal muscle mass has not been examined in details. However, even if muscle atrophy responses are evoked, these are important aspects to consider, as it may contribute to insulin resistance and cascadic effect on normal gastric emptying, as well as important implications for fatigue, workplace performance, and disability compensation. These aspects merit careful observations, studies, and inferences. Some of the alternative interpretations raised in the critique (56) may be important while studying aging populations, with concomitant ongoing sarcopenia and chronic constipation and risk of development of diverticulosis. Interestingly, a recent study has demonstrated that myalgic muscle fibers show dislocated nNOSμ from the sarcolemma but could recover by exercise (74). Whether exercise in general has a positive impact on genomic expression of nNOS and its allosteric regulators are significant and feasible therapeutic avenues to explore.

**Figure 1:**
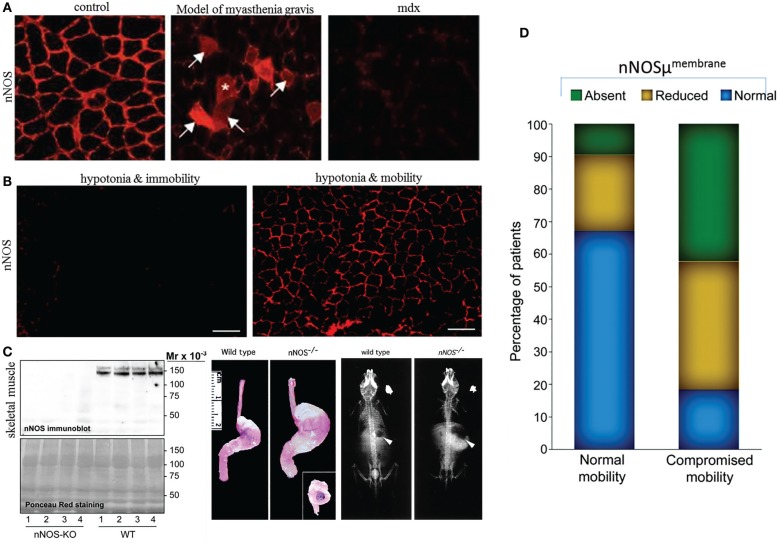
**Sarcolemmal nNOS may be dislocated from membrane in a variety of diseases and may not necessarily be associated with immobility**. **(A)** In contrast to controls, induction of myasthenia gravis result in cytosolic dislocation of nNOS mu. Note loss of sarcolemmal distribution of nNOS in mdx mice, a model for Duchenne muscular dystrophy. Skeletal muscle contains nNOS in different pools, including (i) sarcolemma, (ii) subcellular membranous organelles, (iii) free-floating, (iv) mitochondria, (v) neuromuscular junctions (which is neurophysiologically a fast-conducting synapse, unlike enteric nerve terminal-smooth muscle junctions), and (vi) intrafusal and extrafusal fibers. These different pools are potentially separable by fractionation techniques and complexly interact to impact skeletal muscle mechanical and metabolic actions. **(B)** Skeletal muscle contractile status may not necessarily be correlated with membrane-located sarcolemmal nNOS. Hypotonic muscles show lack or presence of sarcolemmal nNOS, depending on mobility status. These observations merit further detailed history and case-by-case analyses. **(C)** Left panel shows absence of nNOSμ in skeletal muscle extracts in nNOS knockout mice, in comparison to wild-type littermates. This critical observation shows that deletion of exons for nNOSα transcription/translation possesses the necessary molecular capacity to knockout nNOSμ from skeletal muscles. The middle panel shows prominent gastroparesis in nNOS knockout mice. Defective nutrition may potentially impact body weight and skeletal muscle mass and function. The right panel shows fluoroscopic appearance of nNOS knockout mice. Note its small body size in comparison to wild-type littermates. **(D)** Comparative observation from a small-scale study of human skeletal muscle biopsies showing that sarcolemmal nNOS distribution may not have any significant correlation with subject mobility status. Note that in nearly one-third of subjects with normal mobility, sarcolemmal nNOS may be defectively localized; additionally, subjects with impaired mobility may have normal sarcolemmal nNOS [reproduced with permission from Baum et al. (51), Mashimo et al. (52), Finanger Hedderick et al. (53), and Meinen et al. (54)].

There are numerous diseases of the upper esophagus and esophageal body that coexist with skeletal muscle neuromuscular diseases (75). Skeletal muscle disorders can also pose important gastrointestinal motility problems; for example, significant prejunctional vesicular defects as well as smooth muscle atrophy is seen in gut of mdx animal model of Duchenne muscular dystrophy (76, 77). These studies, including my own scant investigations, are in its infancy but definitely warrant aggressive testing at a pilot scale. Skeletal muscle fatigue is a very complex topic and multifactorial in origin. In diabetes, it may potentially result from myosin Va defect, or even from a primary myosin heavy chain defect. It is well-known that diabetes causes significant biochemical dysfunction of myosin heavy chain isoforms, including in skeletal muscles and cardiomyocytes (78, 79). Frankly, I could not comprehend the unreferenced and non-chalant correlation of gastrointestinal motility disorders with patient immobility (56)! Functional GI disorders are a huge clinical problem worldwide, including in the United States (80). The subjects are in distress: think of someone needing to visit the restroom several times a day, with either diarrhea or an incomplete evacuation, or a child with cyclical vomiting, with episodes several times a day. There is a significant loss of physical quality of life, important impact on nutritional status, and loss of workplace efficiency. And, these disorders are not uncommon! Probe an acquaintance with an empathetic ear, and one shall soon hear a story! Most importantly, only scant information is known regarding the pathophysiology of these disorders. For this cohort of patients, who run in millions only within the United States (79), one very important aspect is to provide an answer to “what is my problem?” Most physicians would not endeavor to logically approach and do the complex work up, and often these subjects end up in psychiatric consultations or try alternate methods of medicine. A sort of closure is provided if a logical diagnostic work up is offered. The progression of contents through the gut is a neuromuscular phenomenon, and examining the nerve terminals or the smooth muscles, along with other cellular elements in the neuropil is of paramount importance (31). Despite my personal experience with patients with functional motility disorders for nearly two decades, I have never encountered a single presentation where the subject is incapacitated primarily due to the motility disorder. Of course, someone after a bout of diarrhea or an episode of vomiting may have felt weak, or an elderly person may have been frail, or someone with abdominal pain may have been confined to bed for a few hours, but I have not seen a direct association between long-term GI motility disorder and skeletal muscle fatigue, unless there has been a primary disorder like association of dysphagia in amyotrophic lateral sclerosis. It is interesting and significant that the development of fatigue, for example, in diabetes mellitus, is stochastic, and again, not all skeletal muscles behave in the same way. For example, if my thesis holds well, it still cannot provide a reductionist view of why, despite potential deficiencies of myosin(s) function, we do not commonly encounter diabetes with progressive respiratory failure due to diaphragmatic paralysis (81)! These subtle differences of pathophysiology surely shall lead us into next steps of translational investigations.

Pools of nNOS are in dynamic relation between the membrane and the cytosol, be it in skeletal muscle, cardiomyocyte, or the prejunctional enteric nerve terminal. However, a snapshot of nNOS expression and distribution in skeletal muscles may still be very useful information. A subject could be pursued to provide two samples, at rest and after a period of moderate physical activity in order to examine any potential differences (though developing a protocol for obtaining two biopsies may in itself be a logistic IRB challenge). Furthermore, a wide pool of datasets shall take care of variance, just like in any other experimental setup. Thus, Dr. Percival’s discounting my suggested approaches as “uninformative” (56) is unreliant on critical inferences of the testable hypothesis and not taking into consideration the published evidence related to the differential aspects of nNOS cellular distribution and pathobiology. There are only a few labs worldwide that focus on the molecular medicine of complex GI motility disorders. These investigations should be vigorously pursued as an indefatigable advocacy for motility disorder patients who rightfully may demand to know what exactly the basis of their disease is. As I think through, nNOSμ is present not only in skeletal muscles but also in cardiomyocytes and cavernosal neurons (30, 37). Thus, examining skeletal muscle nNOSμ may not only provide evidence for nNOS synthesis/intracellular transport/localization/activity in the enteric nerve terminals but also provide additional evidence for erectile dysfunction and cardiomyopathy arising from defective nNOSμ signaling in the penile cavernosa and heart. I welcome Dr. Percival’s probing comment (56) who is an expert in the field of nNOSμ in skeletal muscles and invite him to join my endeavors for delivering all things possible to the care of these subjects with recalcitrant disorders that cause enormous suffering. In my opinion, therein lies the true spirit of translational investigations and precision medicine.

## Author Contributions

The author conceptualized, drafted, and approved the final version.

## Conflict of Interest Statement

The author declares that the research was conducted in the absence of any commercial or financial relationships that could be construed as a potential conflict of interest.

## Footnotes

^1^http://www.nature.com/nature/report/index.html?comment=59974&doi=10.1038/464466a
